# ATP13A2 Gene Variants in Patients with Parkinson's Disease in Xinjiang

**DOI:** 10.1155/2020/6954820

**Published:** 2020-11-30

**Authors:** Dan Wang, Hua Gao, Yanxia Li, Sen Jiang, Xinling Yang

**Affiliations:** ^1^Department of Neurology, The Second Affiliated Hospital of Xinjiang Medical University, Urumqi, China; ^2^Department of Neurology, The Fifth Affiliated Hospital of Xinjiang Medical University, Urumqi, China; ^3^Department of Rehabilitation, The Second Affiliated Hospital of Xinjiang Medical University, Urumqi, China

## Abstract

**Objective:**

To analyze the *ATP13A2* gene variants in the Han and Uyghur populations residing in Xinjiang and to determine their correlation with the risk of Parkinson's disease (PD).

**Methods:**

Four *ATP13A2* SNVs—rs56367069 (Arg294Gln), rs151117874 (Thr12Met), rs147277743 (Ala746Thr), and rs2076603—were analyzed in 218 patients (75 Uyghurs and 143 Hans) with sporadic PD and 234 healthy controls (90 Uyghurs and 144 Hans) by Sanger DNA sequencing.

**Results:**

Only one Han patient harbored the AG genotype of the rs147277743 SNV, indicating a frequency of 0.46% in the Han population. In addition, this SNV was not associated with PD risk. The rs2076603 SNV was correlated with PD development, and the A allele in particular was significantly different across ethnicity and age. The rs56367069 and rs151117874 SNVs were not detected in the entire cohort.

**Conclusion:**

*ATP13A2* rs2076603 SNV is associated with PD susceptibility, and the A allele is a PD protective factor in the Han population.

## 1. Introduction

Parkinson's disease (PD) is the second most common neurodegenerative disease after Alzheimer's disease. The incidence rate of PD is about 0.5%-1% among individuals aged 60-65 years and increases up to 1%-3% in those older than 80 years [1, 2]. PD patients exhibit progressive movement disorders such as resting tremor, mask face, underwriting, forward gait, fall-prone kinesthesia, and abnormal posture and gait, as well as complex nonmotor symptoms like psychosis (depression, anxiety, cognitive decline, and hallucinations), autonomic dysfunction (constipation and orthostatic hypotension), sexual dysfunction, sensory symptoms (such as smell, pain, and limbs decreased numbness), and sleep disturbance [3].

The pathogenesis of PD is complicated and influenced by aging, as well as genetic and environmental factors [4]. Recent genome-wide association studies [5–7] have identified several genes, including *α*-synuclein, *LRRK2*, *Parkin*, *DJ-1*, *UCHL1*, *NURR1*, *PINK1*, *FBX07*, *PLA2G6*, *GIGyF2*, *GBA*, and *ATP13A2* (*PARK9*), that are possibly involved in the neuronal degeneration seen in PD. The *ATP13A2* gene in particular is associated with PD susceptibility [8–10] and shows considerable variations in the types and frequencies of mutations in PD patients across different ethnic groups. There are no reports so far of *ATP13A2* gene variants in the PD patients of different ethnic groups in Xinjiang, a geographically and culturally unique region of China. We used Sanger DNA sequencing to analyze the prevalence of four *ATP13A2* single nucleotide variants (SNVs)—rs56367069 (Arg294Gln), rs151117874 (Thr12Met), rs147277743 (Ala746Thr), and rs2076603—in Han and Uygur PD patients in Xinjiang, in order to determine the correlation between *ATP13A2* and PD in these populations.

## 2. Materials and Methods

### 2.1. Subject Cohort

A total of 218 patients (mean age 63.34 ± 10.38 years), including 143 Han (67 males and 76 females) and 75 Uygur (45 males and 30 females), with sporadic PD who were admitted to the Department of Neurology of the Second Affiliated Hospital of Xinjiang Medical University between August 2013 and March 2018 were recruited. All patients met the following inclusion criteria: (1) symptoms in accordance with the diagnostic criteria of UK Parkinson's Disease Society Brain Bank [11], (2) long-term residency of Xinjiang (2 or more generations), and (3) availability of complete clinical data and scores/assessments. The exclusion criteria were as follows: (1) presence of other major Parkinson's syndromes [e.g., secondary parkinsonism and atypical parkinsonian syndromes (Parkinsonism-plus syndrome)], familial Parkinson's syndrome, or hepatolenticular degeneration caused by cerebrovascular disease, trauma, encephalitis, and drugs; (2) comorbidities such as cardiopulmonary dysfunction, hematopathy, malignancy, and liver and kidney dysfunction. In addition, 234 healthy individuals (90 Uyghurs and 144 Hans; 113 males and 121 females; mean age 62.06 ± 9.44 years) were included as controls. The study was approved by the Ethics Committee of Xinjiang Medical University, and all participants provided informed consent.

### 2.2. Methods

Peripheral venous blood (2 ml per subject) was collected into tubes containing 800 *μ*l EDTA. Genomic DNA was extracted using the Blood Genomic DNA Extraction Kit (Tiangen Biotechnology (Beijing) Co., Ltd.) according to the kit instructions. The absorbance (*A*) values of genomic DNA at 260 nm and 280 nm were measured on a UV spectrophotometer using DNA dilution buffer as the control. The absorbance at these two wavelengths indicate the concentration of conjugated bases and proteins, respectively. The DNA sample is acceptable if A260/A280 is 1.7-1.9. The DNA samples were stored at -20°C until further analysis.

#### 2.2.1. Primer Design and Synthesis

The primers were designed based on the ATP13A2 gene sequence available at the National Center for Biotechnology Information (NCBI) public database and in accordance with the principles of primer design and previous studies [12].

#### 2.2.2. PCR Reaction Parameters

Each PCR mix consisted of 3 *μ*l 10× PCR Buffer (Mg2+ plus), 1.5 *μ*l each of the forward and reverse primers (5 *μ*M), 3 *μ*l dNTP Mixture (2.5 mM), 3 *μ*l TaKaRa TaqTM polymerase (5 U/*μ*l), 10-20 ng DNA template, and deionized water to a total volume of 30 *μ*l. The PCR was conducted in the Verity 96-well Thermal Cycler (ABI), and the reaction conditions were as follows: 95°C for 5 min, followed by 15 cycles of 95°C for 30 s, 65°C (-1°C/cycle) for 30 s, and 72°C for 60 s, and 20 cycles of 95°C for 30 s, 52°C for 30 s, and 72°C for 60 s. The obtained PCR product (10 *μ*l) was separated by 2% agarose gel electrophoresis at 120 V for 30 min and examined under an ultraviolet gel imager to confirm that the amplicon was the desired fragment. The reaction conditions for the four sites were essentially similar.

#### 2.2.3. Sanger DNA Sequencing

The PCR products (10 *μ*l per sample) were directly sequenced and aligned with the reference sequence of ATP13A2 gene by Sangon Biotech (Shanghai) Co. Ltd., in order to determine the 4 SNV sites.

### 2.3. Statistical Analysis

All data were analyzed using the SPSS 20.0 software. Numerical data (age) of the two groups were compared using the *t*-test (normal distribution) or median (interquartile range, IQR) (nonnormal distribution) as appropriate. Categorical data (gender and ethnicity) were compared using the *Χ*^2^ test. Gene frequency and genotype distribution were analyzed by the goodness of fit test of the Hardy-Weinberg equilibrium, and the data were expressed as number and frequency (%). Intergroup differences were analyzed using the *Χ*^2^ test and/or Fisher's exact test. Logistic regression was used to analyze the correlation between each SNV and the risk of PD. *P* < 0.05 was considered statistically significant.

## 3. Results

### 3.1. General Information

There were no significant differences in the gender, age, and ethnicities between the case group and the control group (*P* > 0.05) (Table 1). Among the patients, 27 were ≤50 years old and had early-onset PD (EOPD) and 191 were older than 50 years with late-onset PD (LOPD). In the control, there were 33 and 201 individuals, respectively, in the two age subgroups.

### 3.2. Goodness of Fit

The genotype distributions in both the case group and the control group were consistent with the Hardy-Weinberg genetic equilibrium law (Table 2).

### 3.3. ATP13A2 Gene Variant Analysis

The four SNVs of the ATP13A2 gene resulted in distinct PCR-amplified fragments. DNA sequencing revealed the presence of only the GG genotypes for the rs56367069 (Arg294Gln) and rs151117874 (Thr12Met) SNVs in the entire patient and control population, and the AG or AA genotypes were not detected for either SNV. Among the PD patients, only one Han patient with EOPD had the AG genotype of the rs147277743 (Ala746Thr) SNV, while the remaining patients harbored only the GG genotype for this SNV. In contrast, all three AA, AG, and GG genotypes were observed for the rs2076603 SNV, and their distribution frequencies are shown in Table 2.

### 3.4. Genotypic and Allelic Distribution of ATP13A2 SNVs in the Uygur and Han Populations

There were no significant differences in the genotypic and allelic distributions of any of the SNVs between the Uygur patients and healthy controls (*P* > 0.05). For the Han group, the genotypic and allelic distributions of the rs56367069 (Arg294Gln), rs151117874 (Thr12Met), and rs147277743 (Ala746Thr) SNVs were similar between the patients and controls (*P* > 0.05). All the participants were homozygous for the wild-type alleles of rs56367069, rs151117874, and rs147277743. However, significant differences were seen in the frequencies of the rs2076603 genotypes and alleles between the Han patients and healthy controls (*P* < 0.05) (Table 3).

### 3.5. Correlation of the ATP13A2 rs2076603 SNV and PD Susceptibility

#### 3.5.1. Univariate Logistic Regression and Stratified Analysis of Genetic Models of Different rs2076603 Genotypes of the ATP13A2 Gene

In the univariate recessive model, the risk of PD in GG carriers was 0.657 times that of the AG or AA carriers (*P* < 0.05) (Table 4).

#### 3.5.2. Stratified Analysis of rs2076603 Site at ATP13A2 Gene

Further stratification based on ethnicity and age indicated a significant association of PD risk and genotype in Han and older patients.

In the Han recessive model, the A allele is the protective factor of PD, and the risk of PD in GG carriers was 0.522 times than that in AG and AA carriers (*P* < 0.05) and 0.616 times in A carriers compared to the G carriers (*P* < 0.05) (Table 5).

In LOPD, A allele is a protective factor for PD and the risk of PD in A carriers was 0.751 times than that in G carriers (*P* < 0.05) (Table 6).

## 4. Discussion

Although most cases of PD are sporadic, 10%-20% of the patients have a family history of the disease, indicating the role of genetic factors in the pathogenesis of PD. Several novel genes and SNVs associated with PD susceptibility have in fact been identified in recent years, but very few studies have compared these genes between different ethnic groups. Several studies have shown the association of the *ATP13A2* gene with PD susceptibility [8–10]. We examined the prevalence of four SNVs of *ATP13A2* among Han and Uyghur PD patients and healthy individuals in the Xinjiang region and found that the rs2076603 SNV was associated with PD development. The A allele of rs2076603 showed significant differences in frequency across ethnicity and age. In the Han allele model, the risk of PD in A carriers was 0.616 times than that in G carriers, indicating that the A allele is a protective factor for PD in the Han population. Chen et al. identified a new possible pathogenic variant p.Q648X in the ATP13A2 gene of EOPD patients. Patients with pathological ATP13A2 mutations did not respond to levodopa therapy [8]. In LOPD, we found that A allele is a protective factor for PD, and the risk of PD in A carriers was 0.751 times than that in G carriers.

Furthermore, only one patient, a 46-year-old Han female with EOPD, harbored the AG genotype of the rs147277743 (Ala746Thr) mutation, resulting in a frequency of 0.46% for this genotype in the Han population. The patient displayed symptoms like tremors, rigidity, and balance disorder. In addition, she developed severe dyskinesia and wearing-off with the disease progression and could not tolerate pramipexole tablets. After taking 1/4 tablets of Madopar, the patient exhibited dyskinesia that lasted for 30 minutes and resulted in stiffness and inability to exercise. She underwent deep brain stimulation surgery in the subthalamic nucleus at 49 years of age and responded well.

However, due to the small sample size, the AA wild-type genotype could not be detected, and a larger cohort ought to be studied to compare the data of the PD patients carrying different genotypes of this SNV. ATP13A2 mutations can synergistically impair mitochondrial and lysosomal functions [13]. Pathogenic ATP13A2 mutations are loss-of-function mutations leading to alpha-synuclein accumulation [14–16]. Previous studies conducted on PD patients in mainland China and Taiwan have reported only a low frequency of the Ala746Thr and Thrl2Met SNPs in the *ATP13A2* gene [17–24]. Consistent with this, both SNVs were absent in the patients and healthy controls in our study cohort found. The Uyghurs of the Xinjiang region have different genetic lineage [21, 22, 24], lifestyle, and dietary habits compared to the Hans. However, we found no significant differences in the genotypic and allelic distributions of any of the four SNVs between the two ethnic groups. Furthermore, there were no significant differences in the genotypic and allelic frequencies of at least three SNVs between the patients and their corresponding ethnic controls.

There were some limitations in our study that should be considered. First, not all variants were genotyped due to limited funds. Second, multivariate analyses were not performed after adjusting for other risk factors. In addition, our results cannot be replicated in different cohorts due to homogeneity of the sample. Finally, the common genes responsible for themonogenic forms of PD were not screened, and the putative function of the rs2076603 variant was not assessed. Further functional studies are needed to ascertain the pathogenicity of the variants.

To summarize, the rs2076603 SNV of the *ATP13A2* gene is associated with PD development, and the A allele is a protective factor for PD in the Han ethnic group. Further multicenter clinical studies with larger cohort are needed to validate our findings.

## Figures and Tables

**Table 1:** Comparison of general clinical data between PD group and the control group.

	Gender		Age (years)	Ethnic group	
	Female	Male		Han	Uyghur
Case group	106	112	63.00 ± 15.00	143	75
Control group	121	113	62.50 ± 16.00	144	90
*Χ* ^2^/U	0.430		-1.833	0.802	
*P*	0.512^∗^		0.067^∗∗^	0.371^∗^	

Note: ^∗^Pearson chi-square and ^∗∗^median (interquartile range, IQR).

**Table 2:** PD group and control group gene variant test results.

Site of gene	Group	Number of genotype (%)			Number of alleles (%)		HWE (*P*)
rs56367069		GG	AG	AA	G	A	
	Case group	218 (100)	0 (0)	0 (0)	436 (100)	0 (0)	0.999
	Control group	234 (100)	0 (0)	0 (0)	468 (100)	0 (0)	0.999
rs151117874		GG	AG	AA	G	A	
	Case group	218 (100)	0 (0)	0 (0)	436 (100)	0 (0)	0.999
	Control group	234 (100)	0 (0)	0 (0)	468 (100)	0 (0)	0.999
rs147277743		GG	AG	AA	G	A	
	Case group	217 (99.54)	1 (0.46)	0 (0)	435 (99.77)	1 (0.23)	0.972923
	Control group	234 (100)	0 (0)	0 (0)	468 (100)	0 (0)	0.999
rs2076603		GG	AG	AA	G	A	
	Case group	88 (40.37)	93 (42.66)	37 (16.97)	269 (61.70)	167 (38.30)	0.15
	Control group	72 (30.77)	109 (46.58)	53 (22.65)	253 (54.06)	215 (45.94)	0.341

NB: HWE: Hardy-Weinberg equilibrium goodness of fit test.

**Table 3:** Genotype and allele distributions in the case group and control group.

Genotype	Ethnic group	Group	Genotype			Allele	
			GG	AG	AA	G	A
rs2076603	Han	Case group	57	62	24	176	110
		Control group	37	69	38	143	145
		*Χ* ^2^	7.787			8.211	
		*P*	0.02^#^			0.004^#^	
	Uyghur	Case group	31	31	13	93	57
		Control group	35	40	15	110	70
		*Χ* ^2^	0.164			0.027	
		*P*	0.921			0.869	

Note: ^#^*P* < 0.05 when compared with the control group.

**Table 4:** Different genetic models of the *ATP13A2* gene rs2076603 site.

	Dominant ((GG+AG)/(AA))	Recessive ((GG)/(AG+AA))	Allelic (A/G)
Case group	181/37	88/130	167/269
Control group	181/53	72/162	215/253
OR	0.698	0.657	0.731
*P*	0.132	0.033	0.02

**Table 5:** Genetic models of rs2076603 for different ethnic groups.

	Han			Uyghur		
	Dominant ((GG+AG)/(AA))	Recessive ((GG)/(AG+AA))	Allelic (A/G)	Dominant ((GG+AG)/(AA))	Recessive ((GG)/(AG+AA))	Allelic (A/G)
Case group	119/24	57/86	110/176	62/13	31/44	57/93
Control group	106/38	37/107	145/143	75/15	35/55	70/110
OR	0.563	0.522	0.616	1.048	1.107	0.963
*P*	0.05	0.011	0.004	0.91	0.75	0.869

**Table 6:** Genetic models of rs2076603 for different ages.

	EOPD			LOPD		
	Dominant ((GG+AG)/(AA))	Recessive ((GG)/(AG+AA))	Allelic (A/G)	Dominant ((GG+AG)/(AA))	Recessive ((GG)/(AG+AA))	Allelic (A/G)
Case group	24/3	13/14	17/37	157/34	75/116	150/232
Control group	28/5	9/24	29/37	153/48	63/138	186/216
OR	0.7	0.404	0.586	0.69	0.706	0.751
*P*	0.648	0.098	0.164	0.14	0.101	0.048

## Data Availability

The data that support the findings of this study are available on request from the corresponding author. The data are not publicly available due to privacy restrictions.
